# The role of anatomical and functional orientation in identification of parathyroid glands for patients with parathyroidectomy

**DOI:** 10.3389/fendo.2024.1428669

**Published:** 2024-09-30

**Authors:** Peng Zhou, Jing Xu, Yinghao Guo, Lanqing Chen, Yongxiang Liu, Haonan Guo, Changxiu Shao, Qingqing He

**Affiliations:** ^1^ Department of Thyroid and Breast Surgery, the 960^th^ Hospital of People’s Liberation Army, Jinan, China; ^2^ Health company, 92667 Army of PLA, Qingdao, China; ^3^ Jinzhou Medical University, Jinzhou, Liaoning, China

**Keywords:** anatomy, preoperative localization, ectopic parathyroid glands, secondary hyperparathyroidism, parathyroidectomy

## Abstract

**Objective:**

To investigate diagnostic approaches for preoperative localization of secondary hyperparathyroidism, as well as to give surgeons with precise parathyroid gland localization and imaging so that surgery can be performed safely.

**Methods:**

The clinical data of 710 patients with secondary hyperparathyroidism who underwent surgery in our center from October 2009 to October 2023 were retrospectively analyzed. The changes in calcium, phosphorus, and parathyroid hormone levels were observed to ascertain the anatomical location and number of parathyroid glands.

**Results:**

Among the 710 patients, 55 underwent total parathyroidectomy, the others underwent total parathyroidectomy with autotransplantation. In total, 2,658 parathyroid glands were removed, with 43 glands being removed in 35 reoperation cases. The median parathyroid hormone level at 6 months postoperatively was 13.40 (interquartile range, 7.00-29.80) pg/mL. The detection rates of the parathyroid glands before first and repeat surgeries were higher using ^99m^Tc-MIBI SPECT/CT fusion imaging than ultrasound (*P<*0.05). The sensitivity of combined preoperative ^99m^Tc-MIBI SPECT/CT and ultrasound was 92.31%, higher than that of either ^99m^Tc-MIBI SPECT/CT fusion imaging or ultrasound alone (*P* < 0.05). The incidence of ectopic parathyroid glands was 23.8%, and the incidence of ectopic left lower parathyroid glands was 13.2%. The left lower parathyroid gland was the most prone to ectopia.

**Conclusion:**

^99m^Tc-MIBI SPECT/CT fusion imaging, paired with high-frequency ultrasound, can be utilized to diagnose SHPT preoperatively. The most common ectopia site is the left lower parathyroid gland, which is located primarily in the thymus and superior mediastinum. Understanding the functional anatomical distribution of the parathyroid glands is critical for developing effective surgical methods for secondary hyperparathyroidism.

## 1 Introduction

Hyperparathyroidism is a unique clinical disease characterized by either passive or active over-secretion of parathyroid hormone, which disrupts the metabolism of calcium, phosphorus, and bone (1). Hyperparathyroidism is categorized into three kinds based on the cause: primary, secondary, and tertiary. Primary and secondary hyperparathyroidism are prevalent (2). The causes of primary hyperparathyroidism are parathyroid adenoma, hyperplasia, and malignancy (3, 4). Secondary hyperparathyroidism is caused by a variety of conditions that lead to hypocalcemia, prompting the parathyroid glands to adjust by secreting an excessive amount of PTH. It is frequently encountered in situations like renal failure, osteomalacia, and malabsorption syndromes. Tertiary hyperparathyroidism develops from secondary hyperparathyroidism, in which the glands are subjected to extended and severe stimulation, resulting in some hyperplastic tissue changing into adenomas with hyperfunction and autonomously secreting large amounts of PTH. This syndrome is most commonly seen in people with renal insufficiency (5–7).

Parathyroidectomy (PTX) is regarded as the best option for patients who are resistant to medication therapy (8–10). According to research, 5.4%-46.0% of SHPT patients have ectopic parathyroid glands (11), making it difficult for surgeons to precisely locate and count the parathyroid glands. Previous anatomical data on parathyroid glands were mostly obtained from cadaveric study of simulated thyroid surgeries and surgeries for primary hyperparathyroidism, with findings based on histological tests performed following specimen collection (12, 13). However, after autopsy, some parathyroid gland tissues are largely replaced by adipose tissues, altering their distinctive form and color, making identification difficult. Furthermore, differentiating numerous or ectopic parathyroid glands during autopsies is difficult; thus, anatomical data on the number and position of the parathyroid glands are still debated. Patients with SHPT have excessive parathyroid gland growth for a variety of reasons, which increases the volume and weight of the parathyroid glands, making them more ideal for research into anatomical differences in the parathyroid glands. In this study, we collected clinicopathologic data from individuals with SHPT who had PTX. The changes in the anatomical location and number of parathyroid glands were investigated in conjunction with preoperative localization, intraoperative exploration, and postoperative parathyroid hormone (PTH) levels in order to determine the best diagnostic method for parathyroid gland localization.

## 2 Materials and methods

### 2.1 General information

The clinical data of 752 SHPT patients who underwent surgery at the People’s Liberation Army’s 960th Hospital from October 2009 to October 2023 were reviewed. Among them, 14 patients were lost to follow-up, 10 experienced recurrence, and 18 patients were in a persistent state after surgery, therefore they were excluded from this study. Finally, 710 SHPT patients who underwent surgery and had complete clinical data were statistically analyzed, including 292 women and 418 males. The average age was 46.75 ± 11.16 years, with an average dialysis duration of 8.3 ± 4.1 years. Preoperative high-frequency ultrasound and technetium-99m sestamibi single-photon emission computed tomography were performed on all SHPT patients, along with computed tomography scintigraphy fusion imaging. To confirm the removal of the parathyroid glands during surgery, intraoperative fast frozen and postoperative paraffin sections were examined. PTH levels were measured at 20 minutes, one day, three months, and six months after surgery. The hospital’s Ethics Committee accepted the study (Scientific Research Ethics Review No. 2019-03).

### 2.2 Theoretical basis

2.2.1 The starting component responsible for SHPT in patients with uremia is always present. Enlargement and functional augmentation of the parathyroid gland can cause local microscopic morphological alterations, which can be used to assess local anatomy.

2.2.2 Initial surgery with total excision of the parathyroid glands results in PTH levels that are normal or below the reference range. However, in the presence of ectopic or extra parathyroid glands, recurrent hyperparathyroidism (RHPT), or persistent hyperparathyroidism (PHPT), PTH levels will continue to rise. Imaging diagnostics, such as nuclear imaging, have high detection rates, paving the way for further procedures.

2.2.3 To gather correct anatomical data on the parathyroid glands, we retrospectively analyzed patients with SHPT who had successful surgery from a functional anatomical standpoint.

2.2.4 Autopsy results from patients without chronic kidney disease (CKD) found that 2.5%-12.7% of them had tiny parathyroid cell nests in the thymus. In a CKD setting, any continuous stimulation of the parathyroid tissue may result in parathyroid cell nest development, which could be clinically relevant as a cause of recurrent or persistent SHPT (14).

### 2.3 Inclusion and exclusion criteria

Inclusion criteria were as follows: 1) patients with SHPT who received total PTX with autotransplantation (tPTX+AT) or tPTX alone and had normal PTH levels at 1 day postoperatively, with no recurrence during 6 months of follow-up; 2) patients with SHPT who received tPTX + AT or tPTX with a persistent status and reoperation, with PTH levels in the normal range at 1 day postoperatively and no recurrence during 6 months of follow-up.

The exclusion criteria were as follows: 1) patients who did not undergo diagnostic imaging for localization in the persistent state after the initial surgery and did not undergo reoperation; 2) patients who experienced recurrence or were lost to follow-up within 6 months after the first or second surgery; 3) Patients with incomplete records of clinical data.

### 2.4 Diagnostic methods for preoperative localization

#### 2.4.1 Nuclear imaging

The parathyroid glands were examined using the PHILIPS Forte SPECT machine by employing the dual-phase method. ^99m^Tc-MIBI (provided by Beijing Xinkesida Pharmaceutical Technology Co., Ltd.) was intravenously injected, and static imaging was conducted 15 minutes before and 2 hours after injection, followed by SPECT/CT fusion imaging. The low-density area indicates the parathyroid glands.

#### 2.4.2 Ultrasonography

Ultrasonography was performed by an experienced sonologist who has used the same technology to detect parathyroid glands for over ten years. The Hitachi HI VISION Preirus color Doppler ultrasound equipment, equipped with a 7.5-10 MHz linear array probe, was utilized to carefully scan the posterior thyroid region and record the lesions’ locations, sizes, morphology, and blood flow.

### 2.5 Definitions of RHPT and PHPT

RHPT was characterized as a fall in PTH levels to the normal range (<60 pg/ml) within 2 weeks postoperatively, followed by a steady increase in levels lasting more than 6 months.

PHPT was defined as the lowest postoperative PTH level persistently above the upper limit of the normal reference range (>60 pg/ml.) (15).

### 2.6 Definition of ectopic parathyroid glands

The superior parathyroid gland has a rather stable anatomical position. It is placed about 1 cm above the horizontal level of the bottom margin of the thyroid cartilage, near the intersection of the recurrent laryngeal nerve and the inferior thyroid artery on the posterior part of the thyroid gland, and has a diameter of about 2 cm. The inferior parathyroid glands, on the other hand, are more variable and can be found anywhere between the lower one-third of the thyroid gland’s posterior region, anterior to the recurrent laryngeal nerve, and anterior or posterolateral to the thyroid gland’s lower pole surface, extending toward the thymus. Ectopic parathyroid glands are those that fall outside of this range (15, 16).

### 2.7 Regional division of the parathyroid glands

**Figure 1:**
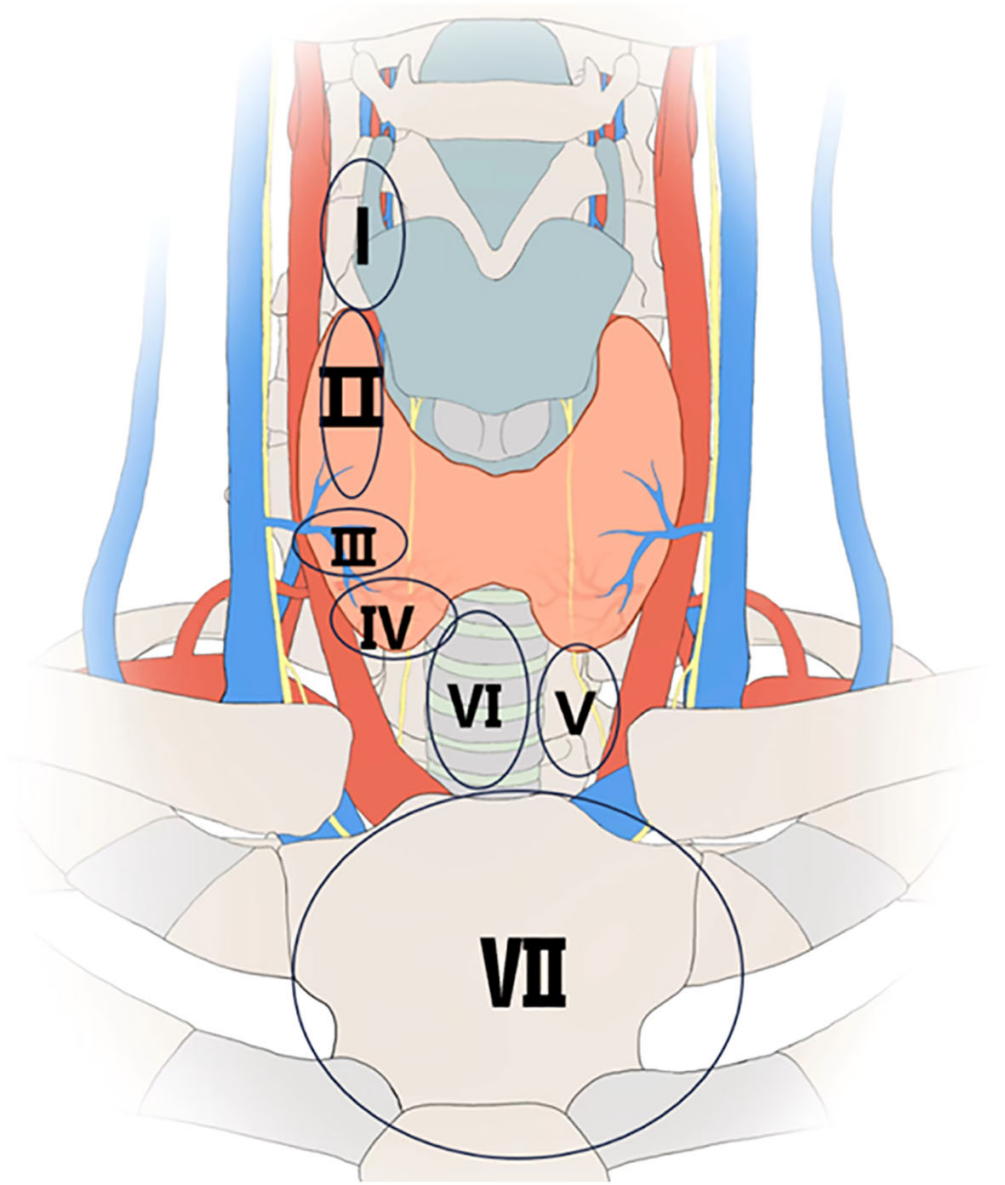
Division of the parathyroid regions.

The distribution of parathyroid glands was divided into seven zones (**Figure 1**).

1. Zone I is defined as the region inside the arterial sheath, above the superior pole of the thyroid gland.
2. Zone II is defined as the region approximately 1 cm above the level of the lower edge of the thyroid cartilage, at the intersection point between the recurrent laryngeal nerve and the inferior thyroid artery, with a diameter of approximately 2 cm.
3. Zone III is defined as the region within 1 cm around the entry of the middle thyroid vein into the thyroid gland.
4. Zone IV is defined as the region located on the posterior one-third of the thyroid gland, anterior to the recurrent laryngeal nerve, and anterior or posterolateral to the inferior pole surface of the thyroid gland.
5. Zone V is defined as the region anterior to the esophagus, between the inferior pole of the thyroid gland and the thymus, including the thymic lingual lobe.
6. Zone VI is defined as the region from the isthmus of the thyroid gland to the sternum, anterior to the trachea.
7. Zone VII is defined as the region posterior to the sternum, in the superior mediastinum (17).

### 2.8 Surgical approach

All patients had heparin-free dialysis the day before surgery. The parathyroid glands were examined intraoperatively using ^99m^Tc-MIBI SPECT/CT and preoperative ultrasonography. 55 individuals received tPTX, while the remainder received tPTX plus AT with 30-60 mg of non-nodular hyperplastic parathyroid tissues. The transplant was carried out on the sternocleidomastoid or pectoralis major muscle. Specimens were subjected to fast pathological evaluation during the operation, followed by standard pathological testing afterward. PTH levels were measured 20 minutes after PTX, and an 80% decrease in PTH levels from the preoperative level suggested surgical success. For patients with PHPT and RHPT, preoperative neck ultrasound and ^99m^Tc-MIBI SPECT/CT were used to identify graft recurrence or missed parathyroid glands during the original surgery, but no parathyroid AT was performed after reoperation.

### 2.9 Postoperative management

Postoperatively, increased calcium intake by bones causes the “hungry bone syndrome,” a severe case of hypocalcemia. Oral administration combined with intravenous injection or infusion pump maintenance is a sort of postoperative calcium supplementation technique, with calcium supplements, vitamin D3, and its analogs given as supportive treatment. The total serum calcium level was kept at 1.8-2.2 mmol/L, and calcitriol shock therapy was given concurrently in the persistent condition. Neck drainage tubes and respiratory tract treatment are comparable to thyroid surgery.

### 2.10 Postoperative follow-up methods and data sources

Follow-up was conducted via telephonic interviews, WeChat, and outpatient visits. Furthermore, data were obtained from the prospectively maintained parathyroid database and medical records, including outpatient, dialysis, postoperative inpatient, surgical, and pathological reports. Preoperative data comprised serum calcium, phosphate, PTH levels, nuclear imaging results, and parathyroid ultrasound results. The number and anatomical location of the removed parathyroid glands were identified by preoperative imaging localization, intraoperative exploration, pathological reports, and postoperative biochemical indicators.

### 2.11 Statistical analysis

The Kolmogorov-Smirnov test was used to determine if continuous variables followed a normal distribution. Normally distributed variables were compared using a t test and expressed as mean ± SD. Otherwise, they were reported as the median (interquartile range), and the Mann-Whitney U test was applied. Categorical variables were compared using Fisher’s exact or chi-square tests based on sample size, and results were reported as frequencies and ratios. Serum calcium was measured using the o-cresolphthalein complexone technique, and the normal range was 2.25-2.75 mmol/L. Parathyroid hormone was quantified using the immunochemiluminescence method, with a reference range of 15-60 pg/ml. The detection rate, sensitivity, specificity, and accuracy of ^99m^TC-MIBI SPECT/CT fusion imaging and ultrasound were evaluated. The detection rate was calculated as (number detected/total number) ×100%, sensitivity as [true positive/(true positive + false negative)]×100%, specificity as [true negative/(true negative + false positive)] ×100%, and accuracy as (true positive + true negative)/(true positive + false positive + true negative + false negative) ×100%. A P-value < 0.05 indicated statistical significance. Data were processed using the Statistical Product and Service Solutions (SPSS) software version 27.0 for Windows (SPSS Inc., Chicago, IL, USA).

## 3 Results

**Figure 2:**
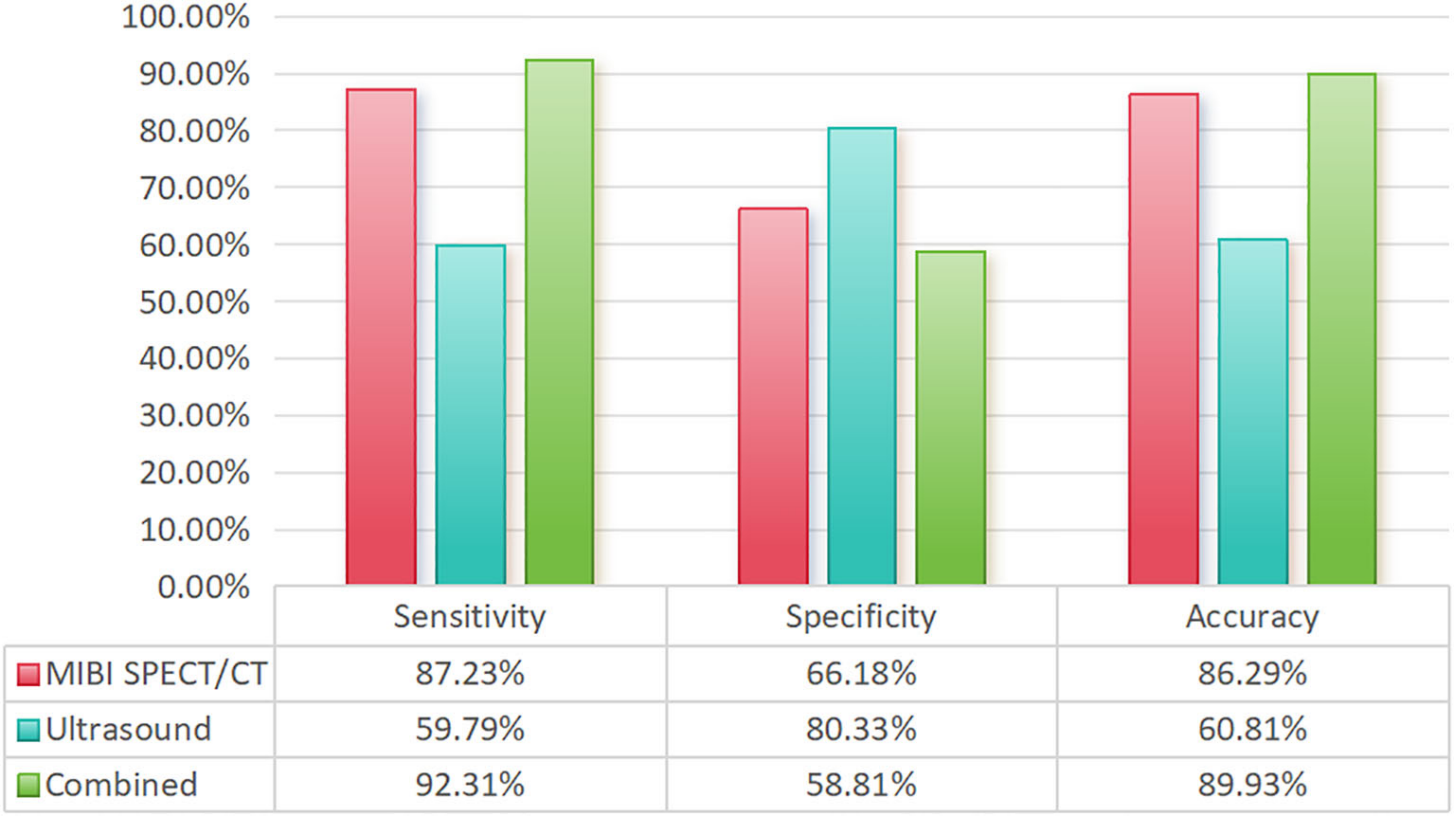
Comparison of the sensitivity, specificity, and accuracy of different examination methods.

55 of the 710 patients received tPTX, while the rest received tPTX with AT. Of them, 675 patients with SHPT had one operation, 32 had two procedures, and 3 had three surgeries. At 6 months postoperatively, the median parathyroid hormone level was 13.40 pg/mL (interquartile range, 7.00-29.80). In total, 2,658 parathyroid glands were removed, including 43 from 35 patients who underwent two procedures. Prior to surgery, ^99m^Tc-MIBI SPECT/CT fusion imaging detected 91.3% of the parathyroid glands, while ultrasound detected 60.0% (P<0.05). Prior to the second surgery, ^99m^Tc-MIBI SPECT/CT fusion imaging had a considerably greater detection rate compared to ultrasonography (P<0.05) (**Table 1**). The sensitivity of ^99m^Tc-MIBI SPECT/CT fusion imaging was 87.23%, while ultrasound was 59.79%. However, ultrasound has a greater specificity (80.33%) than ^99m^Tc-MIBI SPECT/CT fusion imaging (66.18%). Finally, the combined examination had a sensitivity of 92.31%, higher than either test alone (**Figure 2**).

**Table 1:** Comparative analysis of the detection rates of MIBI SPECT/CT fusion imaging and Ultrasound in the first surgery and second surgery.

Group		Detected (glands)	Not Detected (glands)	Detection rate	χ2 value	P value
first surgery	MIBI SPECT/CT	2427	231	91.3%	707.27	<0.001
	Ultrasound	1594	1064	60%		
second surgery	MIBI SPECT/CT	40	3	93.02%	11.01	<0.001
	Ultrasound	26	17	60.47%		

**Figure 3:**
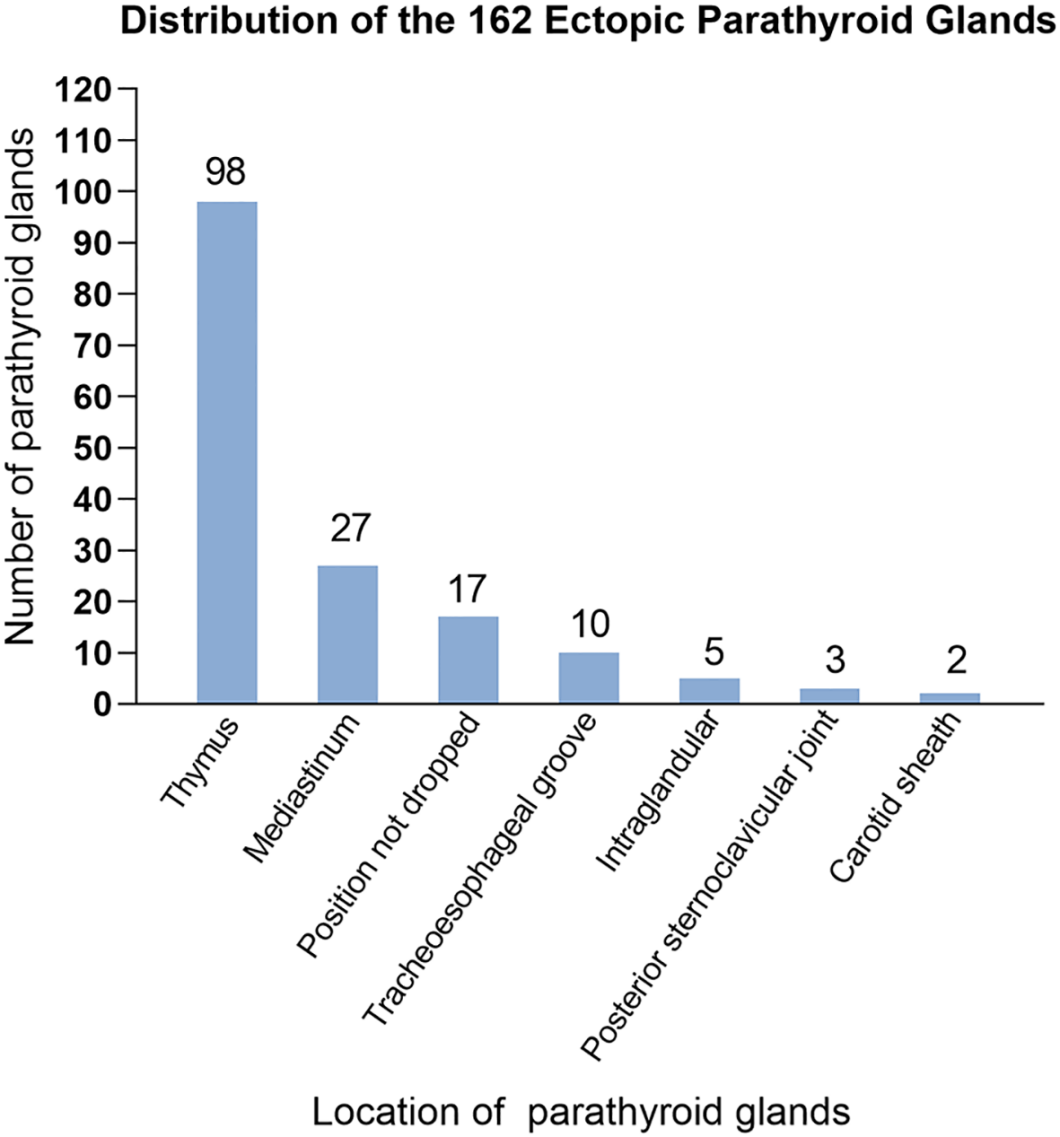
Distribution of the 162 ectopic parathyroid glands.

**Table 2** shows the number and distribution of parathyroid glands in 710 patients with SPTH. The incidence of ectopic parathyroid glands was 23.8%, with the left lower parathyroid gland being more susceptible to ectopia, accounting for 13.2% of instances. In all, 162 ectopic parathyroid glands were found mostly in Zones V and VI. The other sites were the mediastinum, carotid sheath, and thyroid gland (**Figure 3**).

**Table 2:** Number and Distribution of the Parathyroid Glands in 710 Patients with SPTH.

Location	Number	Ectopic Number	Percentage (%)	Ectopic Location
Upper Left	657	11	1.7	Left I
Lower Left	674	89	13.2	Left V, Left VI
Upper Right	698	9	1.3	Right I
Lower Right	659	43	6.2	Left V, Left VI
Other Locations	10		1.4	Anterior to the trachea, carotid sheath, and within the thyroid gland
Total	2698	162	23.8	

**Figure 4:**
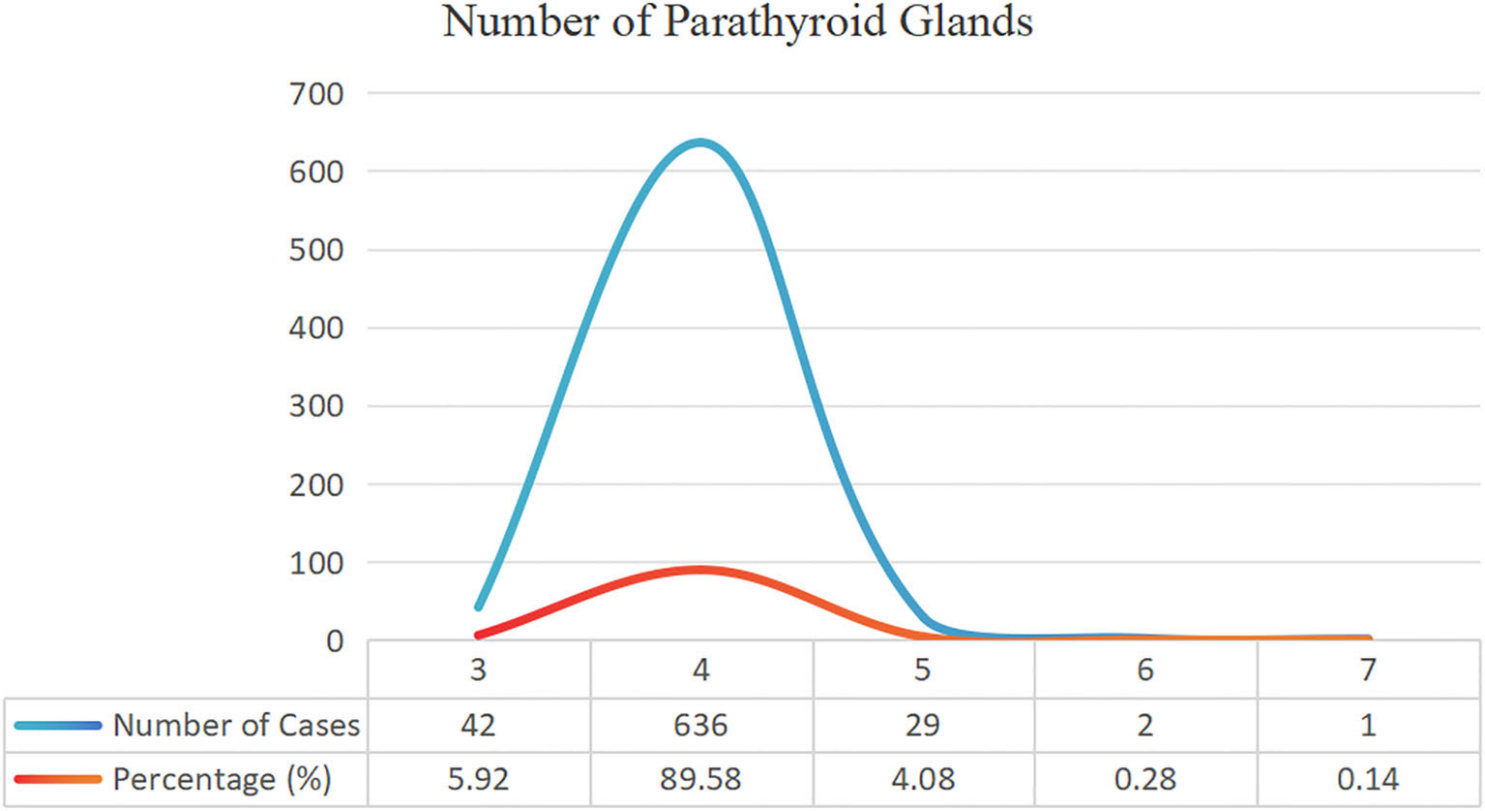
Number of parathyroid glands.

**Table 3** shows the number of parathyroid glands in different patients. Approximately 4.5% of patients had additional parathyroid glands (>4). Of the 35 patients who underwent two surgeries, 43 parathyroid glands were removed. Among them, 10 patients experienced transplant recurrence, 8 experienced missed ectopic parathyroid glands during surgery, 10 had supernumerary parathyroid glands, and 7 had residual glands at the original surgical site (**Figure 4**).

**Table 3:** Number of Parathyroid Glands.

Number	Number of Cases	Percentage (%)
3	42	5.92
4	636	89.58
5	29	4.08
6	2	0.28
7	1	0.14

## 4 Discussion

Surgery remains the most effective treatment option for SHPT patients who have not responded to medication therapies. Choosing the right preoperative diagnostic procedures for localization, assessing changes in the number and location of parathyroid glands, and establishing a rational surgical approach based on the surgeon’s experience is critical for effectively performing PTX and avoiding PHPT or RHPT. Due to an imbalance in calcium homeostasis, patients with SHPT are constantly stimulated by factors such as vitamin D deficiency, hypocalcemia, hyperphosphatemia, and other endocrine disorders, resulting in excessive hyperplasia of all parathyroid glands, with weights and volumes increasing to varying degrees. While the typical weight of a normal parathyroid gland is between 30 and 50 mg, it can exceed 1,690 mg in patients with SHPT (18). PTH levels can be used to objectively assess the presence of residual parathyroid glands after surgery. This physical and functional expansion is better suited for research into the anatomical distribution of the parathyroid glands.

Studies have revealed that compared with the superior parathyroid glands, the inferior parathyroid glands are more prone to anatomical variations in position. The most common sites for ectopic or supernumerary parathyroid glands are the thymus and mediastinum, which was consistent with previous studies (19). During embryonic development, endodermal cells give rise to four parathyroid glands: two superior glands from the fourth pharyngeal pouch and two inferior glands and thymus glands from the third pharyngeal pouch. These glands begin to mature about 5-6 weeks of gestation and go downhill (20). Ectopic parathyroid glands in the bilateral carotid sheaths (0.3%) and bilateral Zone I (3.0%) are typically found in the superior parathyroid glands, which migrate to the dorsal region of the thyroid gland after a short embryonic descent and have a more stable position than inferior parathyroid glands. The inferior glands, on the other hand, descend with the thymus and usually remain around the junction of the inferior thyroid artery and the recurrent laryngeal nerve after they separate from the thymus. They may, however, remain within the thymus tongue and continue their distal migration to other regions, such as the mediastinum (21). We validated this finding in the current investigation. Other ectopic locations include the thymus, anterior mediastinum, tracheoesophageal groove, posterior esophagus, carotid sheath, paralaryngeal space, and thyroid parenchyma. Ectopic glands are predominantly localized in the thymus and anterior mediastinum, and in the current study, we found that the left lower parathyroid gland (13.2%) was the most prone to ectopia, which is consistent with Okada et al.’s findings (22).

Anomalies in the number of parathyroid glands and differences in anatomical position are common causes of prolonged status following SHPT surgery. Previous research has shown that 5.4%-46.0% of patients with SHPT who receive dialysis had ectopic glands (23) and that these ectopic or supernumerary parathyroid glands continue to hypertrophy when stimulated by a uremic environment (24). Although the majority of people have four parathyroid glands, some may have three, five, or more. At our center, we have detected up to seven parathyroid glands, with supernumerary parathyroid glands (more than four glands) accounting for 4.5% of cases. A meta-analysis of 26 studies including 7,005 patients found that 81.4% of patients had four parathyroid glands and 15.9% had anatomical abnormalities, with 11.6% occurring in the neck and 4.6% in the thymus (25). Robert et al. (26) found up to 11 parathyroid glands, with ectopic and supernumerary parathyroid glands occurring in 26% and 16% of patients with SHPT, respectively; the thymus being the most prevalent place. In the current study, we discovered that 6.0% of ectopic parathyroid glands were situated within the thymus, and 48.8% of patients needed reoperation due to the presence of intra-thymic ectopic parathyroid glands. Schneider et al. (21) recommended bilateral thymic tongue lobectomy for patients with fewer than four parathyroid glands in usual locations. Some intramediastinal parathyroid procedures are difficult to conduct through neck incisions, necessitating coordination with thoracic surgeons for endoscopic removal. If the patient’s condition is bad and they are unable to undergo extended concurrent surgery, phased surgery may be chosen based on the clinical benefits. The findings from the current study on 35 patients undergoing reoperation revealed that 10 patients required reoperation due to supernumerary parathyroid glands. Supernumerary and ectopic parathyroid glands make surgery more challenging and raise the risk of RHPT or PHPT.

^99m^Tc-MIBI SPECT/CT fusion imaging in conjunction with high-frequency ultrasound is the most effective preoperative localization diagnostic technique for main or repeat SHPT surgery. In this study, we observed that the ultrasonic sensitivity was 59.79%, which was lower than that reported by Nafisi Moghadam et al. (27) The sensitivity of ^99m^Tc-MIBI SPECT/CT fusion imaging was 87.23%, and the sensitivity of the combined examination was 92.31%, which was consistent with the results of Noda et al. (28) Accurate preoperative parathyroid localization is critical in parathyroid surgery. Color ultrasonography, ^99m^Tc-MIBI SPECT/CT fusion imaging, magnetic resonance imaging, and 11C-choline positron emission tomography/computed tomography are the most often used imaging methods for localization. High-frequency ultrasonography is the most common and required approach for localizing parathyroid glands. However, it is difficult to identify ectopic parathyroid glands below the sternum, as well as those with anterior convexity or posterior thyroid nodules, using ultrasound (29). At the moment, ^99m^Tc-MIBI SPECT/CT fusion imaging is the most useful imaging modality for detecting the parathyroid glands; nevertheless, false positives or negatives may occur due to examiner experience, equipment, thyroid and parathyroid volume, and functional state. In this study, 710 patients with SHPT who had surgical therapy demonstrated that combining the two approaches enhanced the accuracy of preoperative parathyroid localization, which is now the most widely used imaging localization method.

Understanding the anatomy and correctly identifying the location of the parathyroid glands after thyroid surgery reduces problems and improves treatment outcomes. According to studies, the risk of lifelong hypoparathyroidism following total or near-total thyroidectomy ranges between 2% and 33%, with the inferior parathyroid glands typically buried within central compartment lymph nodes (30). In this investigation, we found that ectopic parathyroid glands in the tracheoesophageal groove accounted for 6.2% of cases. During central compartment lymph node dissection, these glands may sustain vascular damage or be accidentally excised. As a result, understanding the anatomical position of the parathyroid glands, combined with meticulous intraoperative fascial dissection and the use of techniques such as nano-carbon negative imaging, can improve parathyroid gland identification and reduce intraoperative injury.

The current study has a few drawbacks. First and foremost, this is a retrospective study, which is prone to selection bias due to its constraints. 18 patients with prolonged postoperative status did not undergo additional imaging examinations or surgical therapy, and these patients and those who lost follow-up may have different anatomical sites and numbers, which may alter the study outcomes. The findings of this study need to be confirmed in a larger sample randomized controlled study. Second, the major participants of this investigation are patients with secondary hyperparathyroidism, but clinical data on people with initial hyperparathyroidism is limited. The next step is to combine the data from patients with primary hyperparathyroidism to confirm the findings of this study.

Finally, ^99m^Tc-MIBI SPECT/CT fusion imaging, paired with high-frequency ultrasound, can be used to diagnose SHPT before surgery. The most common ectopia site is the left lower parathyroid gland, which is located primarily in the thymus and superior mediastinum. Understanding the functional anatomical distribution of the parathyroid glands is critical for developing effective surgical methods for secondary hyperparathyroidism.

## Data Availability

The raw data supporting the conclusions of this article will be made available by the authors, without undue reservation.
